# The Association of Sleep Duration and the Use of Electronic Cigarettes, NHANES, 2015-2016

**DOI:** 10.1155/2020/8010923

**Published:** 2020-02-29

**Authors:** R. Constance Wiener, Christopher Waters, Ruchi Bhandari, Alcinda K. Trickett Shockey, Omayma Alshaarawy

**Affiliations:** ^1^West Virginia University, Department of Dental Practice and Rural Health, School of Dentistry, 104a Health Sciences Addition, PO Box 9415, Morgantown, WV 26506, USA; ^2^West Virginia University, Department of Dental Research, School of Dentistry, 106a Health Sciences Addition, PO Box 9448, Morgantown, WV 26506, USA; ^3^West Virginia University, Department of Epidemiology, School of Public Health, Robert C Byrd Health Sciences Center North, Room G104C, Morgantown, WV 26506, USA; ^4^West Virginia University, Department of Dental Hygiene, School of Dentistry, Robert C Byrd Health Sciences Center North, Room 1192A, Morgantown, WV 26506, USA; ^5^Michigan State University, Department of Family Medicine, College of Human Medicine, Room B113, 788 Service Road, Lansing, Michigan 48824, USA

## Abstract

*Introduction*. The unknown effects of electronic cigarettes are public health concerns. One potential effect of electronic cigarette fluid constituents, such as nicotine, may influence sleep. The purpose of this study is to determine if there is an association between sleep duration and electronic cigarette use.

**Methods:**

A retrospective, cross-sectional study was conducted using National Health and Nutrition Examination Survey (NHANES) 2015-2016. Variables of interest included responses to questions concerning electronic cigarette use, hours of sleep, and other variables associated with sleep. Data analyses were conducted with the Rao-Scott chi square test and logistic regression.

**Results:**

This study was conducted on 2889 participants, aged 18-65 years, of whom 50.7% were female. Using a bivariate analyses of electronic cigarette usage and sleep duration, participants who never used an electronic cigarette were more likely to have appropriate sleep durations as compared with participants who were currently using electronic cigarettes (*P* < 0.0001). After adjusting for sociodemographic variables and cigarette smoking, current electronic cigarette use was associated with higher odds of less sleep duration (adjusted odds ratio = 1.82; 95% CI: 1.18, 2.79; *P* < 0.0001). After adjusting for sociodemographic variables and cigarette smoking, current electronic cigarette use was associated with higher odds of less sleep duration (adjusted odds ratio = 1.82; 95% CI: 1.18, 2.79;

**Conclusions:**

Participants currently using electronic cigarettes are more likely to have less sleep as compared to participants who have never used electronic cigarettes. *Implications*. With sleep time duration being a major factor for proper body function and repair, this study can serve as confirmation that the use of electronic cigarettes is not a harmless health behavior.

## 1. Introduction

Inadequate sleep duration is a public health concern. It is associated with higher all-cause mortality with poor sleep duration [1], higher cancer mortality [2], and increased rates of cardiovascular diseases, diabetes, and obesity [3]. Researchers have related smoking and tobacco use disorder to several sleep disturbances, including poor sleep efficiency, sleep onset latency, number of awakenings [4–6], sleep bruxism, restless leg syndrome [7], and sleep-disordered breathing [8, 9].

The association of sleep and smoking is not fully understood. A nicotine pathway has been postulated as one potential mechanism. It is theorized that the nicotine in tobacco products activates the sympathoadrenal system thereby increasing heart rate, blood pressure, and glucose levels [10]. The activation has a potential to delay or interfere with sleep.

Researchers showed such an effect in preclinical studies where nicotine administration reduced total sleep time duration due to its stimulatory actions [11]. Additionally, researchers found polysomnographic indications of longer sleep onset latency, less total sleep time duration, decreased sleep continuity, and increased wake time among current smokers [9, 12]. Other sleep impairments and insomnia have been noted and associated with the removal of nicotine and its effects during tobacco cessation [12].

Conversely, there are mixed results of sleep disorders and tobacco use in some studies. In a cross-sectional study of young adults, researchers found that sleep quality, sleep time duration, and sleep onset latency were similar among smokers and nonsmokers [13]. In another study, there was no significant correlation of smoking status with apnea hypopnea index, sleep onset latency, and several other measures of sleep quality [14]. When the sample was stratified by age, total sleep time and sleep efficiency were higher and statistically significant in the smoker/former smoker group.

There are few similar studies in which researchers have examined electronic cigarette use and sleep duration. Electronic cigarette devices create an aerosol from a fluid (e-liquid). The e-liquid components can vary immensely. Commercial e-liquids often consist of vegetable glycerin, propylene glycol, proprietary flavorings, and nicotine [15]. The liquids are heated resulting in the formation of an aerosol and are directly inhaled [15]. There were 466 brands and 7764 different flavors of electronic cigarettes identified online from May-August 2012 to December 2013-January 2014 [16]. Many electronic cigarette e-liquids have not been tested. E-liquid fruit flavors, for example, typically contain diacetyl, which has been linked to bronchiolitis obliterans, an irreversible respiratory disease [17]. Some strawberry flavored e-liquids were found to be cytotoxic [17]. There is a concern that vitamins in some e-liquids may cause pulmonary damage [17].

The use of electronic cigarettes has been increasing. Nearly one-fifth (21.6%) of adults, aged 18-24 years, tried electronic cigarettes in 2014 [18]. Approximately 59% of individuals were dual users, reporting both electronic cigarette use and cigarette use [19]. Among high school students, electronic cigarette use increased from 11.7% in 2007 to 20.8% in 2018 [20]. It is important to understand the effect of these products on health.

Currently, there are very few studies on sleep duration and electronic cigarettes, and of the studies, controversy remains as to if an association exists. Researchers recently concluded that dual use of electronic cigarettes with conventional tobacco is associated with decreased sleep quality in women [21]. Other researchers who examined sleep data on 2488 California adolescents found no significant difference in weekday sleep time duration between adolescents who used electronic cigarettes at a higher frequency and lower frequency, although adolescents who used electronic cigarettes slept significantly less on weekends compared with adolescents who did not use electronic cigarettes [22].

It is known that many factors affect sleep duration. In the Unifying Energy Allocation Model of Sleep (the theoretical basis for this study), there are functions that generally occur in wakefulness (including, vigilance, foraging, and reproduction) that are downregulated during sleep; and there are functions that generally occur during sleep (including growth, cellular housekeeping, repair, immune function, and neural network reorganization) and are downregulated during wakefulness [23]. The predominant functions and wake/sleep cycle are altered by drugs/products, alcohol, infection, activity, anxiety/stress, chronic disease, and other factors. In this study, we examined if electronic cigarettes are among the drugs/products that alter sleep duration. However, as this study has a cross-sectional study design, causality cannot be determined. Therefore, questions about the association of electronic cigarette use and sleep remain unanswered. The purpose of this study is to determine the association of electronic cigarette use and sleep time duration among adults covering a wide range of ages (18-65 years). Data are retrieved from the National Health and Nutrition Examination Survey (NHANES) 2015-2016.

## 2. Methods

This study received West Virginia University Institutional Review Board acknowledgement of nonhuman subject research (Protocol number 1907635442). Data used in this study were accessed from the NHANES, 2015-2016, available online at https://wwwn.cdc.gov/nchs/nhanes/ContinuousNhanes/Default.aspx?BeginYear=2015 [24]. The NHANES is a program which began in the 1960s to determine population health based upon approximately 5000 noninstitutionalized U.S. residents. NHANES participants respond to questions concerning demographics, diet, socioeconomic status, and health status. They also undergo dental and medical examinations, laboratory tests, and physiological measurement. The NHANES uses a complex study design to allow for the generalization of findings. Weights and sampling adjustments are provided in the data sets.

The data used in this study were from the Questionnaire Data files of NHANES. The question used for the sleep variable was “How much sleep do you usually get at night on weekdays or workdays?” [24]. The participants' sleep duration responses, in hours, were categorized into “not recommended for their age” and “may be appropriate for their age,” based on the National Sleep Foundation sleep times. For individuals aged 18-25 years, a not recommended sleep duration is <6 hours and >11 hours; for individuals aged 26-64 years, a not recommended sleep duration is <6 hours and >10 hours; and for individuals ≥ 65 years, a not recommended sleep duration is <6 hours and >9 hours [25]. Therefore, “may be appropriate” sleep durations in this study were 6 to 11 hours for individuals aged 18-25 years, 6 to 10 for individuals aged 26-64 years, and 5-9 hours for individuals ≥ 65 years. The National Sleep Foundation indicates 6 hours to *10 or* 11 hours for individuals aged 18-25 years and 5 *or 6* hours to 9 hours for individuals aged ≥65 years in the “may be appropriate” sleep duration category (which slightly overlaps the “not recommended” category) [25].

In NHANES, electronic cigarette use questions were asked in the participant's residence by calibrated interviewers using the Computer-Assisted Personal Interview system. The questions used for the electronic cigarette variable were “Have you ever used an electronic cigarette even one time?” and “During the past 30 days, on how many days did you use electronic cigarettes?” [24]. A negative response to the first question was used to define the participant as a person who never used electronic cigarettes. A positive response to the first question and a response of not having used an electronic cigarette during the past 30 days were used to define the participant as a person who formerly used electronic cigarettes. A positive response to the first question and a response of having used an electronic cigarette within the previous 30 days were used to define the participant as a person who currently uses electronic cigarettes. The authors recognize the complexity of evaluating electronic cigarette use. Whereas never cigarette smoking is generally recognized as fewer than 100 lifetime cigarettes and current cigarette smoking is currently smoking some or every day, similar definitions are not standard for electronic cigarette use [26–28].

In order to more fully describe the sample, electronic cigarette use was further subgrouped by frequency of use over the past 30 days: (1) daily use, (2) intermediate use for more than 5 days to fewer than 30 days, and (3) infrequent use of 1 day to 5 days. Also, to more fully describe the sample, a dual use category was created: (1) current dual use (use of cigarettes *and* electronic cigarettes over the previous 30 days); (2) never dual use (any smoking category (current, former, never) *and* never electronic cigarette use or any electronic cigarette use category (current, former, never) and never cigarette use); and (3) former dual use (smoked >100 lifetime cigarettes but not having used cigarettes over the past 30 days *and* used electronic cigarettes but not having used them over the past 30 days).

**Figure 1:**
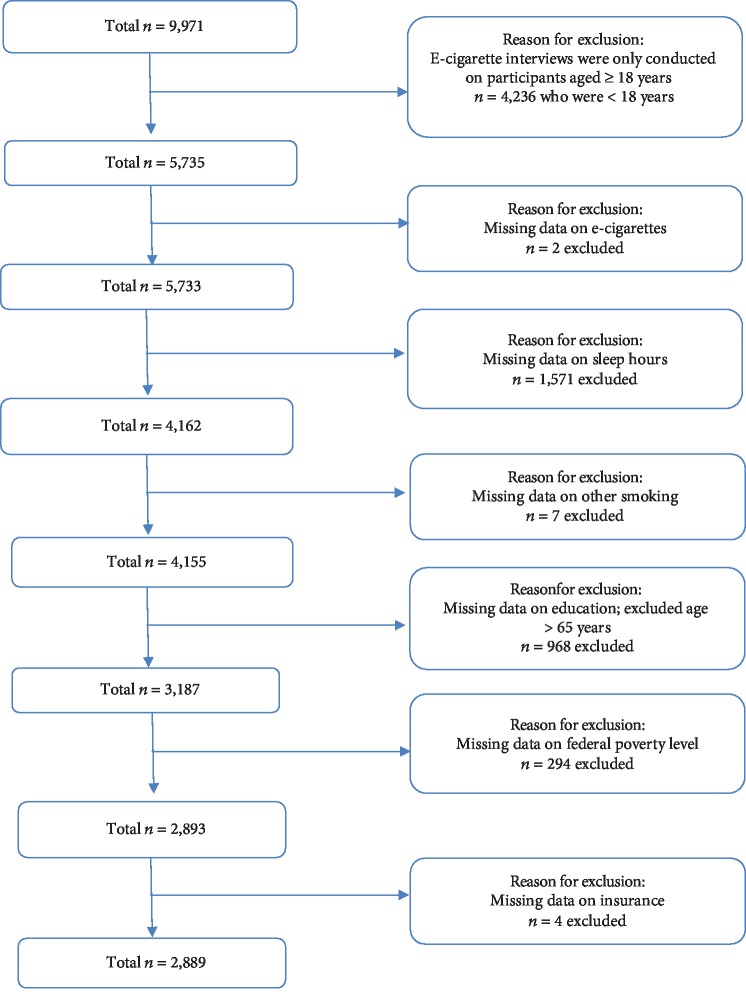
Study sample.

Based on previous research and the Unifying Energy Allocation Model of Sleep, the following variables were associated with both electronic cigarette use and sleep duration and were included in the study: sex (male, female); age (18-25 years, 26-44 years, 45-65 years); race/ethnicity (non-Hispanic white, non-Hispanic black, Mexican American, other); education (high school or less, more than high school); federal poverty level (≤200%, >200%); health insurance (yes, no); cigarette smoking (current, former, never); body mass index (underweight/normal, overweight/obese); alcohol use (no, moderate, heavy, missing); presence of chronic disease defined as arthritis, cardiovascular disease, depression, emphysema, or diabetes (yes, no); and report of daytime sleepiness (0-1/month, 2-4/month, ≥5/month), a measure of sleep quality (Figure 1).

Variables were analyzed for frequency and weighted percentages, presented by current, former, and never electronic cigarette use. Chi-square test was conducted to identify bivariate associations between sleep duration categories (not having recommended sleep duration for their age and may be appropriate sleep duration for their age). The analyses were adjusted for strata, design, and sample weights accounting for the complex sampling design. The key concepts for adequate sample sizes when using NHANES data are that the relative standard error should be less than 30% (standard of the estimate error divided by the value of the estimate) and that the degrees of freedom are calculated by the number of primary sampling units (clusters) minus the number of strata. These criteria limited the number of variables that could be considered in the analyses. Bivariate and multivariable logistic regression analyses were conducted to examine the strength of association of electronic cigarette use and sleep duration. Data analyses were conducted with SAS® version 9.4 (SAS Institute, Inc., Cary, NC). The significance level was set, a priori, at *P* < 0.05.

## 3. Results

The sample description is presented in Table 1. There were 2889 participants, of whom 50.7% were female. There were 18.0% who were aged 18-25 years, 40.0% who were 26-44 years, and 42.1% who were 45-65 years. Most of the participants were non-Hispanic white (60.0%), overweight/obese (60.0%), had no chronic diseases (69.6%), and were >200% of the federal poverty level. There were 21.7% current smokers. Approximately 7.1% of the sample reported currently using electronic cigarettes. Of those participants, 1.5% used electronic cigarettes daily, 1.6% used them intermittently, and 4.0% used them infrequently. About 4.6% of participants had dual electronic cigarette and combustible cigarette use.

Bivariate relationships of electronic cigarette use with the variables of interest are presented in Table 2. Significant relationships with electronic cigarettes were with less sleep duration, older age, less education, lower federal poverty level, lack of health insurance, concurrent cigarette smoking, alcohol use, chronic diseases, snorting, and daytime sleepiness.

Bivariate relationships of sleep duration with the variables of interest are presented in Table 3. Sleep duration was associated with age, race, education, federal poverty level, health insurance, cigarette use, alcohol use, chronic disease, daytime sleepiness, dual use, and electronic cigarette use.

Logistic regression analyses on not having recommended sleep duration for age are presented in Table 4. In the unadjusted logistic regression, participants who reported currently using electronic cigarettes as compared with participants who never used electronic cigarettes had an unadjusted odds ratio of 2.41 (95% CI: 1.66, 3.50; *P* = 0.0010). Participants who formerly used electronic cigarettes had an unadjusted odds ratio of 1.06 (95% CI: 0.73, 1.53; *P* = 0.0861). There was an insufficient sample size to conduct an interaction of participants' electronic cigarette use and cigarette use (3 electronic cigarette use categories × 3 smoking categories).

In a logistic regression model adjusted with demographic variables (sex, age, race/ethnicity, education, federal poverty level, and insurance), the adjusted odds ratio (AOR) for participants who reported currently using electronic cigarettes as compared with participants who never used electronic cigarettes was 2.41 (95% CI: 1.66, 3.50; *P* = 0.0010). Participants who formerly used electronic cigarettes had an adjusted odds ratio of 1.09 (95% CI: 0.76, 1.57; *P* = 0.1291).

In a second model with the addition of smoking, chronic diseases, and alcohol use, the AOR for participants who reported currently using electronic cigarettes as compared with participants who never used electronic cigarettes was 1.82 (95% CI: 1.18, 2.79; *P* = 0.0075). Participants who formerly used electronic cigarettes had an adjusted odds ratio of 0.85 (95% CI: 0.59, 1.24; *P* = 0.0513).

Sample size limitations precluded further analyses based upon frequency of electronic cigarette subgroup categories of intermediate use, infrequent use, current use, and never use. For dual use logistic regression analyses (presented in Table 5), current dual use was associated with not having recommended sleep duration in the unadjusted (OR = 2.95 (95% CI: 1.90, 4.50; *P* = 0.0006)) and both of the adjusted models (AOR = 2.80 (95% CI: 1.68, 4.67; *P* = 0.0041) and AOR = 2.62 (95% CI: 1.65, 4.16; *P* = 0.0010), respectively).

Although not a focus of this study, other factors associated with sleep were also examined. Electronic cigarette use was not associated with snoring (*P* = 0.4828) in bivariate analysis; however, it was associated with snorting (*P* = 0.0034) and daytime sleepiness (*P* < 0.0001). Further analyses with logistic regression failed to reach significance for snorting (OR_e‐cigarette use_ = 0.58 (95% CI: 0.31, 1.07; *P* = 0.3200); AOR_e‐cigarette use_ = 0.60 (0.31, 1.15; *P* = 0.3822); OR_former use_ = 0.60 (95% CI: 0.40, 0.89; *P* = 0.2649); AOR_former use_ = 0.60 (0.35, 1.02; *P* = 0.3135)).

In unadjusted logistic regression, e-cigarette use was more likely to be associated with daytime sleepiness (OR = 0.36 (0.26, 0.52; *P* = 0.0008)) and former e-cigarette use was not (*P* = 0.9219). Further adjusted analyses were not reliable due to the lack of degrees of freedom in computing the results; however, with degrees of freedom set at infinity, daytime sleepiness failed to reach significance (*P* = 0.3845). These results are not presented in a tabular format.

## 4. Discussion

In this study, the researchers report that participants who currently use electronic cigarettes were more likely to have less sleep as compared with participants who never used electronic cigarettes. After adjusting for sociodemographic variables and variables from the Unifying Energy Allocation Model of Sleep, current electronic cigarette use remained associated with higher odds of less sleep duration. As mentioned earlier, the wake-sleep predominant functions are altered by many factors. Our results suggest that electronic cigarette smoking is one such factors; however, as a cross-sectional study, causality cannot be definitively determined by this study: it is possible that electronic cigarette use does impact sleep duration, and it is also possible that when there is reduced sleep duration, there is a greater likelihood of using electronic cigarettes.

In an online search for electronic cigarette use and harm, the first thirty results indicated warnings of dangers associated with electronic cigarettes; further searches described electronic cigarettes as less harmful than smoking, as having a role in tobacco harm reduction. News reports of 2561 hospitalizations and 55 deaths due to lung injuries from electronic cigarettes [29] have increased awareness of the potential dangers of electronic cigarettes.

In support of the potential causal effect, many e-fluids contain stimulants (such as nicotine) that are known to impact sleep. However, there are several electronic cigarette fluids that do not have nicotine in the e-fluids. An alternate possible mechanism for causality is through the link that researchers found between e-liquids with vitamin E acetate and adverse respiratory outcomes. Coughing, airway irritation, and chest pain were associated with electronic cigarette use [29, 30], and such factors may interrupt sleep duration. Although vitamin E acetate seems to be a significant airway irritant, there are many different substances and product sources that are yet to be investigated [29].

Data are scant on electronic cigarette use behavior. Therefore, novel approaches for research on electronic cigarette use behavior are needed. For example, researchers are using genetic analyses to examine tobacco use and other substance use behavior [31]. In a study using Mendelian randomization to calculate genetic correlations and causal effects, researchers found novel genetic correlations between smoking (initiation and cigarettes per day) and reduced sleep behavior [32]. Since the development of the genome-wide association study and the Sequencing Consortium of Alcohol and Nicotine Use, hundreds of novel loci, specifically 564 independent genetic variants assigned to 405 genes, have been discovered to be associated with smoking and drinking behaviors [32]. Genetic analyses between electronic cigarette use and behavioral outcomes may provide similar information concerning genes associated with electronic cigarette use behavior. Unhealthy substance use behavior among adolescents, including tobacco use behavior, declined over the last decade. However, in 2017, approximately 3.6 million U.S. middle school and high school students were using some form of tobacco, selecting electronic vapor products as the most popular product [33]. It is imperative to understand the mechanisms leading to electronic cigarette use.

### 4.1. Strengths and Limitations

The strengths of this study include the national representative sample, the standardized data collection approach, and the ability to adjust for many variables. Though this study has many strengths, there are also certain limitations. NHANES data are observational, and causal inferences cannot be drawn between sleep duration and electronic cigarette use.

The data for sleep duration and electronic cigarette use were self-reported and not objectively measured. The data may be subjected to recall bias. Although sleep can be measured on several parameters, such as sleep time duration, sleep efficiency, sleep onset latency, and the number of awakenings, the researchers considered only sleep time duration in this study. It is possible that the use of a single measure may not fully capture the clinical construct of sleep quality.

Electronic cigarette use was defined as current use (using electronic cigarettes within the previous 30 days), former use (ever having used electronic cigarettes), and never use. This is the conventional method of describing cigarette use; however, the patterns of electronic cigarette use are different from cigarette use and are not standardized for research. Electronic cigarette use is complex and often involves dual use [26–28]. The synergistic effect of electronic cigarettes and conventional tobacco use (dual use) can be much greater than using only one inhalant [34]. The authors provided additional analyses on dual use to address this limitation.

## 5. Conclusion

Participants who currently use electronic cigarettes were 1.82 times more likely not to get their recommended sleep time duration as compared with participants who never used electronic cigarettes. With sleep time duration being a major factor for proper body function and repair, this study can serve as confirmation that the use of electronic cigarettes is not a harmless health behavior.

## Figures and Tables

**Table 1:** Sample characteristics, NHANES 2015-2016, *n* = 2889.

	Number	Weighted percentage
Electronic cigarette use^1^		
Current	191	7.1
Daily use	38	1.5
Intermediate use	35	1.6
Infrequent use	118	4.0
Former	457	17.9
Never	2241	75.0
Cigarette use		
Current use	626	21.7
Former use	480	19.5
Never use	1783	58.8
Dual use^2^		
Current	116	4.6
Former	94	4.2
Never	2679	91.2
Sex		
Female	1496	50.7
Male	1393	49.3
Age in years		
18-25	563	18.0
26-44	1140	40.0
45-65	1186	42.1
Race/ethnicity		
Non-Hispanic white	832	60.0
Non-Hispanic black	669	12.7
Mexican American	904	17.3
Other	484	10.1
Education		
High school/less	1383	37.6
More than HS	1506	62.4
Federal poverty level		
≤200%	1525	39.7
>200%	1364	60.3
Health insurance		
Yes	2233	82.6
No	656	17.4
Body mass index		
Under/normal	1149	40.0
Over/obese	707	60.0
Alcohol use		
No	848	22.7
Moderate	870	36.5
Heavy	720	27.2
Missing	451	13.6
Chronic disease^2^		
Yes	919	30.4
No	1970	69.6
Sleepiness		
0-1/month	1197	37.7
2-4/month	930	34.2
≥5/month	762	28.1
Snoring		
0-2/week	1527	58.3
3-4/week	483	16.4
5 or more times/week	716	25.3
Snorting		
0-2/week	2439	89.1
3-4/week	159	5.6
5 or more times/week	142	5.3
Sleep duration for specific age^3^		
May be appropriate	2505	89.8
Not recommended	384	10.2

Abbreviations: wt%: weighted column percent; HS: high school. ^1^Current use = daily use over the past 30 days; intermediate use = use > 5 days to ≤29 days; infrequent use = ≥1 day to ≤5 days. ^2^Dual use current = smoking cigarettes and using electronic cigarettes over the previous 30 days; former dual use = reporting having smoked >100 lifetime cigarettes and having ever used electronic cigarettes; never = reporting never having smoked >100 cigarettes and reporting never having tried electronic cigarettes. ^3^Chronic disease = positive response to having arthritis, cardiovascular disease, depression, emphysema, or diabetes. ^4^Based upon National Sleep Foundation (https://www.sleepfoundation.org/press-releasenational-sleep-foundation-recommends-new-sleep-times).

**Table 2:** Bivariate analysis of electronic cigarette use with sample variables, NHANES 2015-2016, *n* = 2889.

	Current electronic cigarette use		Former electronic cigarette use		Never electronic cigarette use		*P* value
	Number	Weighted percentage	Number	Weighted percentage	Number	Weighted percentage	
Electronic cigarette use	191	7.1	457	17.9	2241	75.0	
Cigarette use							<0.0001
Current use	116	64.9	254	55.6	256	9.5	
Former use	36	20.4	94	23.2	350	18.5	
Never use	39	14.7	109	21.2	1635	72.0	
Sex							0.0084
Female	76	39.4	218	51.1	1202	51.7	
Male	115	60.6	239	48.9	1039	48.3	
Age in years							<0.0001
18-25	60	28.3	128	27.0	375	14.8	
26-44	80	42.9	186	40.4	874	39.5	
45-65	51	28.9	143	32.6	992	45.7	
Race/ethnicity							0.0604
Non-Hispanic white	77	63.1	186	66.1	569	58.1	
Non-Hispanic black	38	9.6	105	12.1	526	13.2	
Mexican American	52	15.4	106	13.0	746	18.4	
Other	24	11.9	60	8.7	400	10.3	
Education							<0.0001
High school/less	113	57.4	223	40.2	1047	35.1	
More than high school	78	42.6	234	59.8	1194	64.9	
Federal poverty level							0.0002
≤200%	114	53.2	270	48.1	1141	36.4	
>200%	77	46.8	187	51.9	1100	63.6	
Health insurance							<0.0001
Yes	137	76.3	332	76.8	1764	84.6	
No	54	23.7	125	23.2	477	15.4	
Body mass index							0.7431
Under/normal	69	36.7	187	41.8	849	39.9	
Over/obese	121	63.3	267	58.2	1319	60.1	
Alcohol use							<0.0001
No	24	8.8	55	9.7	769	27.1	
Moderate	58	30.3	146	31.9	666	38.2	
Heavy	80	46.3	191	44.6	449	21.3	
Missing	29	14.6	65	13.8	357	13.5	
Chronic disease^1^							0.0053
Yes	76	42.0	164	35.1	679	28.2	
No	115	58.0	293	64.9	1562	71.8	
Sleepiness							<0.0001
0-1/month	46	20.6	133	30.0	1018	41.2	
2-4/month	65	31.9	159	35.2	706	34.2	
≥5/month	80	47.6	165	34.8	517	24.6	
Snoring							0.4828
0-2/week	105	59.5	220	52.1	1202	59.7	
3-4/week	29	15.2	79	18.4	375	16.0	
5 or more times/week	48	25.3	128	29.5	540	24.3	
Snorting							0.0034
0-2/week	151	84.2	372	85.3	1916	90.5	
3-4/week	18	11.0	24	5.7	117	5.0	
5 or more times/week	13	4.8	39	9.0	90	4.5	
Sleep duration for specific age^2^							<0.0001
Not recommended	41	19.9	67	9.9	276	9.4	
May be appropriate	150	80.1	390	90.1	1965	90.6	

Abbreviations: wt%: weighted column percent; HS: high school. *P* values indicate Rao-Scott chi-square test Pr>ChiSq for current/former/never electronic cigarette use. ^1^Chronic disease = positive response to having arthritis, cardiovascular disease, depression, emphysema, or diabetes. ^2^Based upon National Sleep Foundation (https://www.sleepfoundation.org/press-releasenational-sleep-foundation-recommends-new-sleep-times).

**Table 3:** Bivariate analysis of sleep duration with sample variables, NHANES, 2015-2016, *n* = 2889.

	Not recommended sleep duration for specific age^1^		May be appropriate sleep duration for specific age^1^		*P* value
	Number	Weighted percent	Number	Weighted percent	
Electronic cigarette use					<0.0001
Current	41	13.9	150	6.3	
Former	67	17.3	390	18.0	
Never	276	68.8	1965	75.7	
Cigarette use					<0.0001
Current use	132	35.5	494	20.1	
Former use	56	16.5	424	19.8	
Never use	196	48.0	1587	60.0	
Dual use^3^					<0.0001
Current	Cell size suppressed^4^	10.8	87	3.9	
Former	Cell size suppressed	2.8	85	4.3	
Never	Cell size suppressed	86.4	2333	91.8	
Sex					0.3620
Female	196	48.7	1300	51.0	
Male	188	51.4	1205	49.0	
Age in years					0.0009
18-25	48	11.0	515	18.7	
26-44	152	41.3	988	39.7	
45-65	184	47.7	1002	41.5	
Race/ethnicity					<0.0001
Non-Hispanic white	82	45.2	750	61.6	
Non-Hispanic black	138	25.7	531	11.3	
Mexican American	114	19.7	790	17.0	
Other	50	9.4	434	10.2	
Education					<0.0001
High school/less	225	57.8	1158	35.3	
More than high school	159	42.2	1347	64.7	
Federal poverty level					<0.0001
≤200%	251	61.1	1274	37.2	
>200%	133	38.9	1231	62.8	
Health insurance					0.0328
Yes	286	78.4	1946	83.1	
No	98	21.6	558	16.9	
Body mass index					0.0910
Under/normal	167	44.2	982	39.6	
Over/obese	209	55.8	1498	60.4	
Alcohol use					<0.0001
No	116	26.9	732	22.2	
Moderate	90	23.5	780	38.0	
Heavy	111	33.9	609	26.5	
Missing	67	15.7	384	13.4	
Chronic disease^1, 2^					<0.0001
Yes	176	46.8	743	28.5	
No	208	53.2	1762	71.5	
Sleepiness					<0.0001
0-1/month	122	25.8	1075	39.1	
2-4/month	116	33.9	814	34.2	
≥5/month	146	40.3	616	26.7	

Abbreviations: wt: weighted; HS: high school. ^1^Based upon National Sleep Foundation (https://www.sleepfoundation.org/press-releasenational-sleep-foundation-recommends-new-sleep-times). ^2^Chronic disease = positive response to having arthritis, cardiovascular disease, depression, emphysema, or diabetes. ^3^Dual use current = smoking cigarettes and using electronic cigarettes over the previous 30 days; former dual use = reporting having smoked >100 lifetime cigarettes and having ever used electronic cigarettes; never = reporting never having smoked >100 cigarettes and reporting never having tried electronic cigarettes. ^4^Numerical entries into the cell categories are suppressed for participant confidentiality.

**Table 4:** Logistic regression of electronic cigarette use on not recommended sleep duration for specific age^1^, NHANES 2015-2016, *n* = 2889.

	Unadjusted			Model 1			Model 2		
	Unadjusted odds ratio	95% CI	*P* value	Adjusted odds ratio	95% CI	*P* value	Adjusted odds ratio	95% CI	*P* value
Electronic cigarette use									
Current use	2.41	1.66, 3.50	0.0010	2.41	1.52, 3.82	0.0042	1.82	1.18, 2.79	0.0075
Former use	1.06	0.73, 1.53	0.0861	1.09	0.76, 1.57	0.1291	0.85	0.59, 1.24	0.513
Never use	1.00^∗^			1.00			1.00		
Sex									
Male				1.11	0.93, 1.34	0.2387	1.13	0.91, 1.40	0.2750
Female				1.00			1.00		
Race/ethnicity									
NHB				2.69	1.93, 3.74	<0.0001	2.67	2.01, 3.57	<0.0001
Mexican American				1.19	0.75, 1.88	0.2862	1.23	0.83, 1.82	0.3074
Other				1.16	0.73, 1.84	0.2687	1.21	0.80, 1.85	0.3480
NHW				1.00			1.00		
Age in years									
26-44				2.24	1.36, 3.69	0.0687	2.08	1.36, 3.20	0.0102
45-65				2.82	1.81, 4.42	<0.0001	2.20	1.43, 3.37	0.0014
18-25				1.00			1.00		
Education									
High school or less				1.93	1.39, 2.68	0.0007	1.72	1.29, 2.30	0.0003
More than high school				1.00			1.00		
Insurance									
No				0.83	0.59, 1.18	0.2814	0.83	0.61, 1.11	0.2004
Yes				1.00			1.00		
Federal poverty level									
≤200%				2.19	1.39, 3.43	0.0021	1.94	1.25, 3.02	0.0032
>200%				1.00			1.00		
Yes							1.67	1.27, 2.19	0.0002
No							1.00		
Alcohol use									
Heavy use							1.68	1.14, 2.46	0.1564
No							1.68	1.09, 2.60	0.2090
Moderate							1.00		

Abbreviations: CI: confidence interval; NHB: non-Hispanic black; NHW: non-Hispanic white. ^∗^Reference groups are identified as having an odds ratio of 1.00. ^1^Based upon National Sleep Foundation (https://www.sleepfoundation.org/press-releasenational-sleep-foundation-recommends-new-sleep-times). ^2^Chronic disease = positive response to having arthritis, cardiovascular disease, depression, emphysema, or diabetes. Model 1 is adjusted for sex, race/ethnicity, age, education, insurance status, and federal poverty level. Model 2 is adjusted for sex, race/ethnicity, age, education, insurance status, federal poverty level, smoking status, chronic disease status, and alcohol use. A missing indicator was created for alcohol use (result not presented for missing category).

**Table 5:** Logistic regression of dual use ^1^ on not recommended sleep duration for specific age^2^, NHANES 2015-2016, *n* = 2889.

	Unadjusted			Model 1			Model 2		
	Unadjusted odds ratio	95% CI	*P* value	Adjusted odds ratio	95% CI	*P* value	Adjusted odds ratio	95% CI	*P* value
Dual use									
Current use	2.95	1.90, 4.58	0.0006	2.80	1.68, 4.67	0.0041	2.62	1.65, 4.16	0.0010
Former use	0.70	0.29, 1.69	0.0525	0.86	0.37, 2.02	0.1415	0.86	0.37, 1.97	0.1492
Never use	1.00^∗^			1.00			1.00		
Sex									
Male				1.12	0.93, 1.36	0.2229	1.14	0.92, 1.41	0.2279
Female				1.00			1.00		
Race/ethnicity									
NHB				2.68	1.91, 3.76	<0.0001	2.71	2.02, 3.63	<0.0001
Mexican American				1.18	0.74, 1.88	0.2881	1.20	0.80, 1.82	0.2610
Other				1.15	0.71, 1.86	0.2687	1.20	0.77, 1.87	0.3334
NHW				1.00			1.00		
Age in years									
26-44				2.16	1.32, 3.54	0.0698	2.10	1.36, 3.24	0.0090
45-65				2.66	1.72, 4.11	<0.0001	2.19	1.42, 3.38	0.0016
18-25				1.00			1.00		
Education									
High school or less				1.94	1.40, 2.70	0.0007	1.78	1.32, 2.40	0.0002
More than high school				1.00			1.00		
Insurance									
No				0.84	0.58, 1.20	0.3038	0.84	0.61, 1.15	0.2805
Yes				1.00			1.00		
Federal poverty level									
≤200%				2.18	1.38, 3.44	0.0024	2.00	1.31, 3.06	0.0013
>200%				1.00			1.00		
Chronic disease^3^									
Yes							1.73	1.32, 2.26	<0.0001
No							1.00		
Alcohol use									
Heavy use							1.76	1.19, 2.59	0.0803
No							1.63	1.07, 2.49	0.2596
Moderate							1.00		

Abbreviations: CI: confidence interval; NHB: non-Hispanic black; NHW: non-Hispanic white. ^∗^Reference groups are identified as having an odds ratio of 1.00. ^1^Dual use categories: current dual use = use of cigarettes *and* electronic cigarettes over the previous 30 days; never dual use = any smoking category (current, former, never) *and* never electronic cigarette use or any electronic cigarette use category (current, former, never) and never cigarette use; former dual = having smoked >100 lifetime cigarettes but not having used cigarettes over the past 30 days *and* having used electronic cigarettes but not having used them over the past 30 days. ^2^Based upon National Sleep Foundation (https://www.sleepfoundation.org/press-releasenational-sleep-foundation-recommends-new-sleep-times). ^3^Chronic disease = positive response to having arthritis, cardiovascular disease, depression, emphysema, or diabetes. Model 1 is adjusted for sex, race/ethnicity, age, education, insurance status, and federal poverty level. Model 2 is adjusted for sex, race/ethnicity, age, education, insurance status, federal poverty level, chronic disease status, and alcohol use. A missing indicator was created for alcohol use (result not presented for missing category).

## Data Availability

The data used to support the findings of this study are available from the corresponding author upon request.
